# A Multidisciplinary Standardized Patient Simulation for Using Trauma-Informed Care for Pregnant Patients

**DOI:** 10.15766/mep_2374-8265.11474

**Published:** 2024-11-26

**Authors:** Danielle Nichole Olson, Anna Brandt, Sarah Greywitt, Kelly S. Gibson

**Affiliations:** 1 Third-Year Maternal Fetal Medicine Fellow, Division of Maternal Fetal Medicine, Department of Obstetrics and Gynecology, the MetroHealth System and Case Western Reserve University School of Medicine; 2 Simulation Specialist, Simulation Institute, the MetroHealth System and Case Western Reserve University School of Medicine; 3 Maternal Fetal Medicine Division Director, Division of Maternal Fetal Medicine, Department of Obstetrics and Gynecology, the MetroHealth System and Case Western Reserve University School of Medicine; † Co-second author

**Keywords:** Motivational Interviewing, Obstetrics, Pregnancy, Trauma-Informed Care, Women's Health, OB/GYN, OB/GN - Maternal & Fetal Medicine, Quality Improvement/Patient Safety, Simulation, Standardized Patient, Well-Being/Mental Health

## Abstract

**Introduction:**

Pregnant patients with prior traumatic experiences and their providers face challenges during prenatal care, peripartum, and postpartum. To date, no structured simulations have been published focused on improving care for patients in subsequent pregnancies.

**Methods:**

This multidisciplinary simulation included trainees and providers. Standardized patients were used. The patient was multiparous with a remote history of substance use and was initiating prenatal care late due to concerns related to the prior traumatic experience of losing custody of a newborn after a Department of Children and Family Services report had been opened in a prior pregnancy. Simulation participants completed the prenatal intake and counseling regarding this prior experience. Debriefing sessions reviewed critical actions, including collecting a history, empathizing with the patient, praising efforts to maintain pregnancy health, discussing available resources, constructing a plan for care, and utilizing motivational interviewing techniques. The simulation and debriefing sessions were allotted 30 minutes total. Pre- and postsimulation surveys evaluated for increased comfort and knowledge in caring for patients with prior traumatic experiences.

**Results:**

Simulation participants included obstetrics and gynecology students and residents, nurses, advanced practice providers, generalist attendings, and maternal fetal medicine fellows and attendings. Sixty participants completed the presimulation survey. Twenty-seven (45%) completed the postsimulation survey. Responses were paired for analysis. Scores on knowledge assessments improved postsimulation (*p* = .001). Responses suggested increased comfort in discussing prior traumatic experiences (*p* = .13).

**Discussion:**

This simulation led to improved background knowledge and comfort regarding providing trauma-informed care during pregnancies impacted by a prior traumatic event.

## Educational Objectives

By the end of this activity, learners will be able to:

1. Practice strategies to establish rapport when caring for a patient with a prior traumatic experience.
2. Practice ways of beginning conversations with patients with a prior traumatic experience.
3. Identify and discuss how patients’ past experiences can affect current pregnancies and subsequent engagement with medical care.
4. Identify essential elements of motivational interviewing.

## Introduction

Trauma is defined as a single event or series of events resulting in a lasting impact on one's mental, social, physical, or emotional state.^1,2^ These events can span from physical abuse and natural disasters to prior poor experiences with the health care system. Patients with prior traumatic experiences often experience higher rates of mental health conditions, chronic physical symptoms, and retraumatization when interacting with the health care system, leading to decreased utilization of health care services and subsequent adverse outcomes.^3,4^

Trauma-informed care is defined as a constellation of tools and approaches to aid providers in offering adequate medical care while creating an environment of physical and emotional safety for survivors, reducing the risk of retraumatization for their patients, promoting healing, and empowering patients by preserving their sense of control.^2,5,6^ Practicing trauma-informed care leads to a strengthened partnership between the patient and the provider, enhancing the patient's engagement in their medical care and improving outcomes.^7,8^

Team-based simulations have long been a tool used in medical education, providing unique opportunities to learn about and understand rare and complex scenarios.^9,10^ The understanding and application of trauma-informed care are increased through training as well.^11,12^ Few studies have been conducted evaluating the use of simulation in trauma-informed care; those few have been primarily limited to applications in human trafficking and adverse childhood experiences, and no studies to date have evaluated these concepts in a reproductive health care setting.^13–15^ Providers of reproductive health care should strengthen the implementation of trauma-informed care through training clinicians and learners in what it means to be trauma informed and how to best provide this care to a wide array of patient experiences.^5^ Team-based simulation and education on trauma-informed care enable trainees to increase their knowledge and familiarity in caring for patients with prior adverse experiences.

We designed a multidisciplinary educational simulation with the goal of teaching reproductive health care workers, enabling them to gain knowledge and skills in caring for patients with prior traumatic experiences through a formative feedback exercise. Members of the simulation team included providers from maternal fetal medicine (MFM) and perinatal social work with extensive experience in providing trauma-informed care in pregnancy. Standardized patients were used to maintain realism of counseling and simulate building of rapport. Participants included medical students, residents, fellows, generalist and subspecialty attendings, registered nurses, nurse managers, and advanced care providers who routinely worked with pregnant patients. To our knowledge, this simulation is the first of its kind.

## Methods

### Development

Annually, the department of obstetrics and gynecology at our tertiary care center hosts a citywide simulation day that health care providers from many disciplines can attend to learn and practice complex topics in a simulation setting. Our multidisciplinary simulation was designed to include trainees and providers routinely caring for pregnant patients and was utilized in the annual simulation conference in October 2023.

### Equipment/Environment

No simulated medications, equipment, or laboratory values were used. An exam room was simulated using a classroom with chairs for providers and an exam table. A simulation case caveat was read to the participants prior to entry into the simulated exam room; no printed materials were provided.

### Personnel

One standardized patient was used and provided through the simulation center at this tertiary care center. The standardized patient was educated by the simulation case authors using printed materials, as was the standard for this simulation center. Education materials included information detailed in Appendix A; a succinct overview of the character and scripted responses to possible prompts were provided as detailed in Appendix B.

One or two simulation facilitators were used for each run of the simulation. The facilitators also led the debriefing sessions. Facilitators were practitioners who did not participate in the simulation. Training materials for the simulation facilitators included a list of possible actions that would dictate case flow (Appendix C), as well as a facilitator education guide (Appendix D). This guide included background information on the simulation case as well as educational material on motivational interviewing, adverse coping mechanisms after a traumatic experience, and trauma-informed care principles. The guide was used to direct informative discussion during debriefing sessions as well. Prior to each run of the simulation, the facilitators were given a new debriefing sheet to note critical actions taken by the participants.

The vital participant roles to be assigned were one nurse, one trainee (medical student or resident), and one attending. The remaining participants were assigned as additional nurses or trainees.

### Implementation

The simulation was designed in collaboration between the MFM department and the Simulation Institute at the MetroHealth System with the intent to be included in the annual simulation conference. After completion of the simulation protocol, the case flow (Appendix E) was tested by simulation staff prior to the simulation conference.

On the day of the conference, the simulations took place in classrooms rearranged to model physical exam rooms that would be used in a clinic setting. The participants were divided into small groups, and a presimulation briefing session was conducted, introducing participants and facilitators. There was no distribution of educational materials or discussion of educational content prior to the simulation. Roles were assigned and the case caveat read, as detailed in the debriefing form (Appendix F).

The simulation then began and was permitted to proceed for approximately 15 minutes, following the case flow as detailed in Appendix E. Participants interviewed and counseled the patient as a group to approximate a usual prenatal care intake appointment and ensure allotment of time for all participants to complete the case. Various elements of counseling provided yielded increasing levels of engagement by the patient, as detailed in Appendix C. If participants encountered difficulties and did not proceed in a timely fashion, the facilitators were allowed to give assistance and prompts to ensure forward flow. Each learner participated in the simulated case one time.

A critical action checklist was created for the simulation (Appendix F). This list included the essential medical components of providing prenatal care as well as the individualized aspects of delivering trauma-informed care. Special attention was given to listing components of motivational interviewing as critical actions as well. The simulation was completed after all critical actions had been completed or after the time had elapsed if the critical actions were unable to be completed.

After the simulation ended, the facilitators conducted a debriefing session using the standardized debriefing form (Appendix F). This debriefing session spanned approximately 15 minutes, reviewing decision-making and the case flow of the simulation, opportunities for improvement, and the educational content described above (Appendix D). Participants were then welcome to give feedback on their experiences with the simulation and to ask questions of the facilitators. This feedback was not formally evaluated.

### Debriefing

The debriefing form (Appendix F) was used to guide conversation and education after the simulation. This form included a summary of the objectives of the simulation, scoring for each of the critical actions for the simulation, key points of discussion for the debriefing session, and an outline of motivational interviewing tactics to review with simulation participants. Data from debriefing forms were not collected, as these forms were constructed to guide conversation, not to serve as an evaluation measure.

During debriefing, participants were asked about elements of the case they believed they had performed well, as well as elements they believed were opportunities for improvements. Participants were also asked various ways to approach a patient with the presented history, as well as ways they could adjust these approaches if elements of the history were changed. Each critical action was then reviewed, including any notes taken by simulation facilitators indicating areas for improvement.

### Learner Assessment

To determine the benefits of this simulation, we created an anonymous survey using REDCap to be distributed pre- and postsimulation (Appendices G and H). Unique links were distributed by email. Responses were anonymous. Following the simulation, if a presimulation survey had been completed, a paired anonymous postsimulation survey link was distributed by email. We chose to use a survey to encourage comprehensive feedback on knowledge and comfort level changes, to be inclusive of all learner types, and to reduce bias. The survey included a cover letter granting consent to use response data in publication and quality improvement upon completion of the survey. This project was undertaken as a quality improvement initiative and as such did not constitute human subjects research; it therefore was granted exemption by the institutional review board.

Contents of the survey included demographic information, questions regarding experience and comfort in caring for patients with a prior traumatic experience, and background knowledge imperative to providing trauma-informed care. This background knowledge assessment included questions relating to how patients’ prior experiences might impact their engagement with the medical system, delineations of the role of each member of the health care team in trauma-informed care, details and strategies to employ in trauma-informed care, questions relating to mental health conditions in patients with a prior traumatic experience, and motivational interviewing skills. The survey was distributed to all simulation participants. If a presimulation survey had been completed, a new link to the same survey was created to be performed postsimulation. Responses from participants who completed both versions of the survey were paired for analysis to assess for improvement. Nominal data were analyzed using McNemar tests. Continuous data were analyzed using paired-sample *t* tests. Responses on Likert-scale questions were analyzed using binomial sign tests.

## Results

Health care providers from multiple systems throughout the city were invited to take part in the simulation. One hundred fourteen participants across four hospital systems registered to participate. The demographic information of the participants is detailed in Table 1. Presimulation surveys were distributed to these individuals, 60 of whom (53%) completed them. For all participants who responded, the average number of years in practice was 13.1 (*SD* = 11.5 years). Fifteen participants (25%) were registered nurses, 19 (32%) were residents, 10 (17%) were generalist attending physicians, three (5%) were MFM fellows, three (5%) were advanced practice providers, five (8%) were medical students, four (7%) were nursing managers or supervisors, and one (2%) was an MFM attending physician. Most participants (28, 47%) detailed their practice as both inpatient and outpatient, with fewer reporting primarily outpatient practice (25, 42%) or primarily inpatient practice (seven, 12%).

**Table 1.**
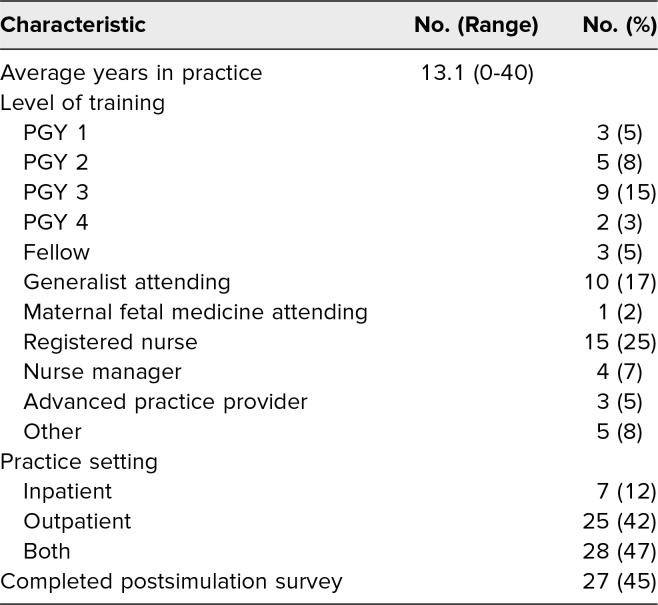
Simulation Participants’ Demographics (*N* = 60)

Participants’ prior experiences are detailed in Table 2. The majority of participants reported having previously cared for a patient with a history of a traumatic event (49, 82%). A higher percentage of participants reported they had personally witnessed a traumatic patient experience that they believed had potential to impact future pregnancies (58, 97%).

**Table 2.**
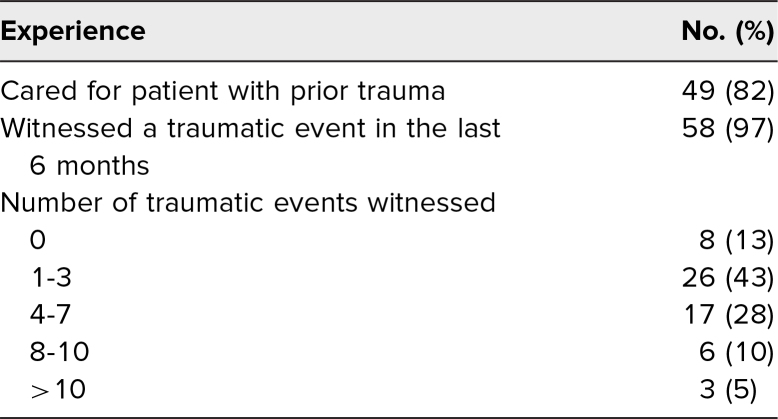
Simulation Participants’ Prior Experience (*N* = 60)

Postsimulation surveys were distributed to those who had completed a presimulation survey. The response rate was 45%, with 27 postsimulation surveys being completed. For the simulation evaluation analysis, responses on completed pre- and postsimulation surveys were paired, and data from those who did not complete a postsimulation survey were discarded. These results are detailed in Table 3. Composite scores of knowledge assessments improved postsimulation (*p* = .001), with an average score increase of 7%. Postsimulation, 22 of the 27 respondents (82%) were able to describe essential elements of motivational interviewing, compared to 19 (70%) presimulation. Presimulation, 15 (56%) answered that they believed they could only provide trauma-informed care if they understood the details of the patient's trauma, improving to five of the 27 (18%) postsimulation.

**Table 3.**
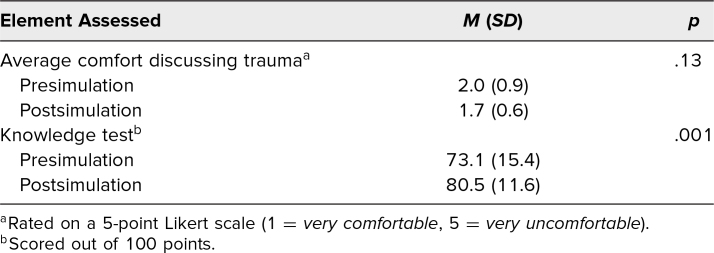
Survey Score Changes After Simulation (*n* = 27)

The survey also inquired about change in providers’ level of comfort in communication while caring for patients with a prior traumatic experience. These results are detailed in Table 3. Postsimulation, these scores suggested improvement, though this did not reach statistical significance (*p* = .13).

The simulation has not yet been repeated to date.

## Discussion

When a pregnant patient has a history of a prior traumatic experience, they and their providers face unique challenges. The American College of Obstetrics and Gynecology states that “it is important for obstetrician–gynecologists and other health care practitioners to recognize the prevalence and effect of trauma on patients and the health care team and incorporate trauma-informed approaches to delivery of care.”^5^ Given the complexities involved in pregnancy, childbirth, and the postpartum period, it is essential for providers working in these areas to practice trauma-informed care when caring not just for a patient with a history of a traumatic event but for all patients.^5,6^ Significant strides have been made in recent years to destigmatize maternal mental health conditions and to emphasize the importance of trauma-informed care; however, many areas for improvement remain.

There is a paucity of literature dedicated to trauma-informed prenatal care and how to apply its principles. To date, no simulation protocols have been published aiming to improve the care provided to pregnant patients with a prior traumatic experience. A significant portion of the health care providers who completed the surveys noted that they had cared for a patient with a prior traumatic experience. An even larger portion reported that they had witnessed a traumatic event that could potentially affect a future pregnancy. This highlights the need for specific training in how to identify patients who may have had a prior traumatic experience and how to best provide these patients with the standard of care.

Our simulation, while novel in its goals, also aimed to include a multidisciplinary group of providers. During pregnancy, a patient can encounter many types of providers with varied levels of training. It is essential that each of these team members be trained to provide trauma-informed care. Across the multiple levels of training involved in our simulation, participants demonstrated improved knowledge of trauma-informed care after completing the session. Additionally, many felt more comfortable discussing the details of a patient's prior traumatic experience, an essential element in building rapport with this vulnerable population.

Like every educational tool, this simulation has limitations. First, it was performed at a large academic hospital with specialized and dedicated simulation and clinical staff during a protected educational time, resources that may not always be available across health care systems. Second, survey completion rates were suboptimal for this intervention. While the majority of individual elements assessed in the pre- and postsimulation surveys showed improvement after the intervention, the power was not sufficient to evaluate each of these separately, limiting the ability to make fine adjustments to the protocol. Lastly, as a survey-based method was used to evaluate the simulation, the results may be subject to response bias.

This simulation led to improved background knowledge and comfort in providing trauma-informed care during pregnancies impacted by a prior traumatic event. The simulation will be added to the regular curriculum at our tertiary care hospital. By publishing, we wish to share the format so that the simulation can be used in other systems to improve trauma-informed prenatal care.

## Appendices


Standardized Patient Case.docxStandardized Patient Guide.docxFacilitator Notes.docxFacilitator Education Guide.docxCase Flow.docxDebriefing Form.docxTrauma-Informed Care Presurvey.docxTrauma-Informed Care Postsurvey.docx
*All appendices are peer reviewed as integral parts of the Original Publication.*
