# Supplementary material for: A Multidisciplinary Standardized Patient Simulation for Using Trauma-Informed Care for Pregnant Patients
Source: MedEdPORTAL. 2024 Nov 26;20:11474. doi: 10.15766/mep_2374-8265.11474 (PMC11590754; doi:10.15766/mep_2374-8265.11474)
Supplement: Supplementary file 1 — Standardized Patient Case.docxStandardized Patient Guide.docxFacilitator Notes.docxFacilitator Education Guide.docxCase Flow.docxDebriefing Form.docxTrauma-Informed Care Presurvey.docxTrauma-Informed Care Postsurvey.docx [file mep_2374-8265.11474-s001.zip › C. Facilitator Notes.docx]

Appendix C: Facilitator Notes

*To be used as a quick reference of case flow for the facilitator during the simulation.*

| Facilitator Notes - Changes and CASE Branch Points | | |
| --- | --- | --- |
| **Intervention / Time Point** | **Change in Case** | **Additional Information** |
| Providers begin to collect history | n/a | Patient acts disengaged, giving few details and offering short responses to questions. |
| Patient is asked why she is presenting to prenatal care late in pregnancy | Prompts patient response | Patient states: “I’ve just been really busy and nervous about stuff.” |
| Patient is further prompted regarding reported nervousness. | Prompts patient response | Patient states: “I just don’t want to go through that again.” |
| Providers express empathy with patient’s experiences | Prompts patient response | Patient discloses details of last pregnancy and delivery. |
| Social work consultation and additional resources are offered. | n/a | Patient accepts these and becomes more engaged in conversation. |
| Recommendations are made to complete lapsed prenatal care. | n/a | Patient expresses readiness and is amenable to ultrasound/labs/other orders. |
| Providers should summarize the plan for the patient, including resources accepted and plans for prenatal care. | n/a | Patient thanks the team for their care. |
| If providers do not summarize care, simulation not yet completed. | Prompts patient response | Patient states: “I’m overwhelmed by all of this information.” |
