# Supplementary material for: A Multidisciplinary Standardized Patient Simulation for Using Trauma-Informed Care for Pregnant Patients
Source: MedEdPORTAL. 2024 Nov 26;20:11474. doi: 10.15766/mep_2374-8265.11474 (PMC11590754; doi:10.15766/mep_2374-8265.11474)
Supplement: Supplementary file 1 — Standardized Patient Case.docxStandardized Patient Guide.docxFacilitator Notes.docxFacilitator Education Guide.docxCase Flow.docxDebriefing Form.docxTrauma-Informed Care Presurvey.docxTrauma-Informed Care Postsurvey.docx [file mep_2374-8265.11474-s001.zip › D. Facilitator Education Guide.docx]

Appendix D: Facilitator Education Guide

*To be used during Simulation Facilitator education and during the educational session after the simulation.*

**Module Summary**

This is a communication-based simulation involving a patient with a prior poor experience in her last pregnancy. Anticipated learners include Obstetric Nurse, Residents, Fellows, and Attendings. An Anesthesiologist may also attend. The case involves a patient who had DCFS involvement in a prior pregnancy and does not have custody of this child. Learners must recognize the patient’s concerns in this new pregnancy and discuss resources, reassure the patient, and form a plan.

The facilitator(s) will brief the learners and then present the patient information and start the scenario. Roles will be assigned. The primary assigned nurse and medical trainee will begin the interaction. The assigned attending physician and possible additional nurses/trainees will be present in the room, and will participate as needed/requested by the assigned primary nurse and medical trainee. The learners will conduct the patient interaction, executing the critical actions necessary to achieve the objectives. Following the simulation, the facilitator(s) will conduct a debriefing, working with the learners to explore decision-making, aspects of care that went well, and opportunities for improvement.

Approximately 15 minutes should be allotted for the simulation, with an additional 15 minutes following for education and debriefing.

**Content for Post-Simulation Educational Discussion**

Motivational interviewing is a method of developing/communicating trust during conversation. The AAFP developed the acronym OARS to outline the essential components of this strategy.

- Ask **O**pen-ended questions: Instead of using “yes” or “no” questions, open-ended questions foster conversation and elicit the patient’s thoughts. If action is needed, such as substance use cessation, this allows for the patient to originate the idea, significantly increasing the chances of success.
- Offer **A**ffirmations: When a patient is successful in meeting a goal, praise their efforts. When the patient is not successful in meeting a goal, express empathy, and ongoing support. This builds trust and normalizes struggling to maintain positive change, thus the patient is more likely to discuss these concerns with their provider.
- Practice **R**eflective listening: Allowing the patient to feel “heard” followed not by telling them what to do with a concern, but rather dissecting and discussing and their thoughts, helping them arrive at a solution.
- **S**ummarize the visit: Recap the conversation, including the patient’s concern(s), current status of these, and plan moving forward for each problem. This should be an active discussion, allowing the patient to clarify and add details where necessary. Goals can be identified in this step, with an outlined plan for when to discuss their progress.

Possible signs of inadequate coping patients with a prior traumatic experience may exhibit:

- Traumatic re-experiencing: flashbacks, nightmares.
- Avoidant behaviors, numbness or detachment.
- Guilt, self-blame, or extended unhealthy hyper-focus on circumstances of case.
- Vulgar language, irritability, poor interpersonal style.
- Stress or burnout that is impairing care and function.
- Increased anxiety, panic, withdrawal, low mood.
- Physical symptoms such as poor sleep, severe fatigue, severe headaches.

Trauma-Informed Care principles

- Trauma is described as events or circumstances experienced by an individual as physically or emotionally harmful or life-threatening, which can result in adverse effects on the individual’s functioning and well-being.
- Trauma-informed care describes multiple tactics to help healthcare providers care for vulnerable patients, reduce re-traumatization, and promote healing for patients with prior poor experiences.
- Many frameworks exist, but the common principles are as follows:
  - Safety
    - Create a space where the patient, support persons, and healthcare staff feels safe physically and psychologically.
    - Feeling safe helps to decrease the sense of threat, and allows all members of the care team, including the patient, to prioritize formulating a future plan of care.
  - Trustworthiness and transparency
    - An open discussion of goals for each step of the care plan should be had, allowing the patient to ask clarifying questions.
    - Establishing a rapport with the patient and building trust is essential. Each patient has unique perspectives, and respecting these perspectives strengthens the therapeutic relationship.
  - Peer support and mutual self-help
    - Discussion with a patient that others may have similar lived experiences, and allowing for them to support and be supported by their peers may offer a unique addition to a patient’s care.
  - Collaboration and mutuality
    - Ask and assess how the patient and any family members are coping.
    - Every member of the care team is responsible for providing trauma-informed care, from the front desk administrative task to the attending physician. Having a united, team-based approach and shared message creates a sense of collaboration that allows the patient to feel they are well supported.
  - Empowerment, collaboration, and choice
    - Trauma often involves a loss of sense of power, and many who experience trauma feel a degree of helplessness when faced with experiences that may be re-traumatizing.
    - Allowing patients to play an active part in decision making regarding their care, and involving them in the discussion of how to approach the potential obstacles they may face helps to give agency and a sense of choice.
    - Reinforce with the patient that they are the most essential member of their care team.
  - Acknowledgement of cultural, historical, and gender issues
    - Multiple elements of a patient’s background can contribute to how adverse experiences affect them and their outlook on future medical care. Understanding potential known and unknown biases can help individualize the response to a patient’s past trauma and allow for a unique approach which will best support their needs.
    - It is important to remember that specific traumatic events may impact some patients more than others, therefore approaching the patient rather than the history is essential.
