# Supplementary material for: A Multidisciplinary Standardized Patient Simulation for Using Trauma-Informed Care for Pregnant Patients
Source: MedEdPORTAL. 2024 Nov 26;20:11474. doi: 10.15766/mep_2374-8265.11474 (PMC11590754; doi:10.15766/mep_2374-8265.11474)
Supplement: Supplementary file 1 — Standardized Patient Case.docxStandardized Patient Guide.docxFacilitator Notes.docxFacilitator Education Guide.docxCase Flow.docxDebriefing Form.docxTrauma-Informed Care Presurvey.docxTrauma-Informed Care Postsurvey.docx [file mep_2374-8265.11474-s001.zip › E. Case Flow.docx]

**Appendix E: Case Flow**

*To be used as a reference for the expected flow of the simulated case.*

Team members are identified, roles are noted.

↓

Team members are read the delivery synopsis and patient background as above.

↓

The scenario begins and the team is able to begin collecting a history on the patient.

↓

Initially, the patient is hesitant to discuss her last pregnancy in detail, but as the provider expresses empathy with her concerns, she begins to confide her concerns in the team.

↓

Providers should empathize with the patient and discuss each of her concerns.

↓

Providers should give reassurance and praise of the patient’s efforts to become sober and motivation to stay sober for the health of her pregnancy.

↓

Providers should discuss resources available to the patient if she desires.

↓

A plan to complete up to date prenatal care should be made.

↓

Lastly, the visit should be summarized with the patient.

↓

The simulation is completed.
