# Supplementary material for: A Multidisciplinary Standardized Patient Simulation for Using Trauma-Informed Care for Pregnant Patients
Source: MedEdPORTAL. 2024 Nov 26;20:11474. doi: 10.15766/mep_2374-8265.11474 (PMC11590754; doi:10.15766/mep_2374-8265.11474)
Supplement: Supplementary file 1 — Standardized Patient Case.docxStandardized Patient Guide.docxFacilitator Notes.docxFacilitator Education Guide.docxCase Flow.docxDebriefing Form.docxTrauma-Informed Care Presurvey.docxTrauma-Informed Care Postsurvey.docx [file mep_2374-8265.11474-s001.zip › F. Debriefing Form.docx]

**Appendix F: Critical Action Review, Debriefing Form**

*To be used as a tool during the debriefing and educational session.*

Case Caveat: Patient is a 26-year-old G2P1001 at 28w0d by sure last menstrual period presenting to the office to establish prenatal care. She is approximately 15 minutes late for her scheduled appointment.

She has a history of one prior full term vaginal delivery in an outside hospital system, for which you cannot see records. She reports she is overall feeling well today, no complaints. The remainder of her medical, surgical, social, and family history is uncomplicated.

Learner Objectives

1. Practice strategies to establish rapport when caring for a patient with a prior traumatic experience
2. Practice ways of beginning conversations with patients with a prior traumatic experience
3. Identify and discuss how patients’ past experiences can affect current pregnancies and subsequent engagement with medical care
4. Identify essential elements of motivational interviewing

Critical Action Checklist

| Action | Met | Partially Met | Not Met |
| --- | --- | --- | --- |
| 1. Collect and address the patient’s relevant history. |  |  |  |
| 2. Empathize with her past experiences as they relate to this pregnancy. |  |  |  |
| 3. Praise patient’s efforts to maintain a healthy pregnancy. |  |  |  |
| 4. Offering social work/resources in a sensitive manner. |  |  |  |
| 5. Ensure plan to get ultrasound and lab work completed. |  |  |  |
| 6. Ask open-ended questions. |  |  |  |
| 7. Practice reflective listening. |  |  |  |
| 8. Summarize the visit. |  |  |  |

Key points

- Past adverse pregnancy events and outcomes may serve as a significant source of trauma in future pregnancies.
- Prior birth trauma may result in an array of challenges, including late/inadequate engagement in care.
- Patients with poor experiences require individualized care; empathy and support are paramount.
- Motivational interviewing tools are important in assessing a patient’s readiness to change, support and plans to maintain said change.

Debriefing questions

- Can one or more participants summarize the case? What happened and how did you respond?
- What similarities did you notice between this case and experiences you have had with similar situations with your own patients?
- Can each participant describe an element of the case that they think went very well?
- Can each participant describe an element of the case that they think could have gone differently or could be improved for future interactions?
- What did you learn that will impact how you interact with patients in similar situations in the future?
- Is there anything that you would change about this activity to strengthen its impact for future learners?

Notes:
