# Supplementary material for: A Multidisciplinary Standardized Patient Simulation for Using Trauma-Informed Care for Pregnant Patients
Source: MedEdPORTAL. 2024 Nov 26;20:11474. doi: 10.15766/mep_2374-8265.11474 (PMC11590754; doi:10.15766/mep_2374-8265.11474)
Supplement: Supplementary file 1 — Standardized Patient Case.docxStandardized Patient Guide.docxFacilitator Notes.docxFacilitator Education Guide.docxCase Flow.docxDebriefing Form.docxTrauma-Informed Care Presurvey.docxTrauma-Informed Care Postsurvey.docx [file mep_2374-8265.11474-s001.zip › H. Trauma-Informed Care Postsurvey.docx]

**Appendix H: Trauma-Informed Care Post-Survey**

*To be used as a post-simulation survey to evaluate for change in knowledge/comfort of simulation participants.*

Attendees of the 2023 MetroHealth Perinatal Conference,

We look forward to you joining us for the 23rd annual Perinatal Conference!

Attached is a post-simulation survey. We ask that you take 5-10 minutes to complete this. We strive to make this conference the best that it can be, and your responses are incredibly helpful to achieving that goal. By completing this survey, you would be granting your permission to use this data for publication and quality improvement processes not only confined to MetroHealth and Cleveland, but to the medical education field as a whole. Your unique link is below.

Thank you so very much for your time.

1. What is your level of training?
   1. PGY 1
   2. PGY 2
   3. PGY 3
   4. PGY 4
   5. Fellow
   6. Attending, generalist
   7. Attending, MFM
   8. Attending, anesthesiology
   9. Registered Nurse
   10. Nurse Manager/PCC
   11. Advanced Practice Provider
   12. Other
2. How many years have you been in practice (including training years)?

__________

1. In what setting do you primarily practice?
   1. Inpatient
   2. Outpatient
   3. Both
2. Have you ever experienced a traumatizing or distressing patient encounter?
   1. Yes
   2. No
3. Approximately how many distressing patient encounters have you experienced in the last 6 months?
   1. 0
   2. 1-3
   3. 4-7
   4. 8-10
   5. >10
4. How likely are you to recognize symptoms of distress in yourself?
   1. Very likely
   2. Somewhat likely
   3. Neutral or unsure
   4. Somewhat unlikely
   5. Very unlikely
5. How likely are you to recognize symptoms of distress in others?
   1. Very likely
   2. Somewhat likely
   3. Neutral or unsure
   4. Somewhat unlikely
   5. Very unlikely
6. Have you cared for patients with a prior traumatic experience with the healthcare system in a previous pregnancy?
   1. Yes
   2. No
7. How comfortable do you feel discussing how a patient’s prior traumatic experiences affects their current pregnancy?
   1. Very comfortable
   2. Somewhat comfortable
   3. Neutral or unsure
   4. Somewhat uncomfortable
   5. Very uncomfortable
8. A patient safety assessment is only indicated if a provider has specific concerns.
   1. True
   2. **False**
9. Patients with uncontrolled PTSD, depression, and/or anxiety are at an increased risk for obstetric complications in subsequent pregnancies.
   1. **True**
   2. False
10. Patients with a prior traumatic experience are often hesitant to engage in routine medical care.
    1. **True**
    2. False
11. What are some appropriate strategies to approaching a patient with a history of a prior traumatic experience? (Select all that apply.)
    1. **Listening and empathizing with the patient’s lived experience**
    2. Avoiding discussing the topic to avoid unnecessary re-traumatization
    3. **Individualizing the approach to the patient’s medical care**
    4. **Discuss available resources with the patient**
    5. Immediately involving social work or similar entity at the first prenatal visit
12. What is motivational interviewing?
    1. A method to motivate patient to follow a recommended course of treatment
    2. **A patient-centered, directive method for enhancing intrinsic motivation to change by exploring and resolving the patient’s feelings and thoughts on a topic**
    3. A method of discussing the risks/benefits of various medical treatments
    4. A tool used to entice patients to disclose elements of their history
    5. A systems-based approach to identify factors contributing to population health
13. What principles are important in motivational interviewing? Please select all that apply.
    1. **Resist the urge to tell the patient what to do**
    2. **Understand the patient’s motivations**
    3. **Listen and empathize**
    4. **Empower the patient**
    5. **Ask open-ended questions**

For objective queries, correct answers are depicted in **bold font.**
