# Supplementary material for: A Multidisciplinary Standardized Patient Simulation for Using Trauma-Informed Care for Pregnant Patients
Source: MedEdPORTAL. 2024 Nov 26;20:11474. doi: 10.15766/mep_2374-8265.11474 (PMC11590754; doi:10.15766/mep_2374-8265.11474)
Supplement: Supplementary file 1 — Standardized Patient Case.docxStandardized Patient Guide.docxFacilitator Notes.docxFacilitator Education Guide.docxCase Flow.docxDebriefing Form.docxTrauma-Informed Care Presurvey.docxTrauma-Informed Care Postsurvey.docx [file mep_2374-8265.11474-s001.zip › B. Standardized Patient Guide.docx]

**Appendix B: Standardized Patient Guide**

*To be used as a quick reference for the Standardized Patient.*

**Background**

You are a 26-year-old at 28 weeks pregnant. You are presenting for your first prenatal care visit. Your last pregnancy, you struggled with opioid use and were unable to successfully stabilize on suboxone therapy. You actively relapsed the day you delivered a full-term baby girl. Protective services were involved given the substance use, and custody was not granted to you. Your cousin currently has custody of your daughter, who is now 3. Since then, you have quit opioids “cold turkey” and are determined to stay sober for this pregnancy, for which you hope to maintain custody. Given your past experiences, interacting with prenatal care has made you very nervous and you have not been able to initiate prenatal care due to this trauma.

**Volunteered history**

Your last delivery was out of state, you cannot remember the hospital name. You were 9 months pregnant at the time and went into labor at home. You came to the hospital and delivered about 10 hours after. The delivery was uncomplicated from what you recall.

**History if asked**

DCFS was called when you were admitted in your last pregnancy. You were actively using at the time. You did consent to a tox screen on admission which was positive. DCFS ultimately did not grant you custody of your daughter. You left the hospital the day after delivery, but otherwise there were no complications during your admission.

**Scripted Responses**

If asked why you are presenting to prenatal care so late, reply “I’ve just been really busy and nervous about stuff.”

If prompted further regarding what you are nervous about, reply “I just don’t want to go through that again.”

If social work consultation and additional resources are offered, be accepting of these and become engaged. Can reply “I’d be okay with talking to the social worker” or “I can try that” if other resources are offered.

If asked about your prior pregnancy, reply “My last pregnancy, I was still using. I tried really hard to get on Subutex, but it didn’t work. I tried going cold turkey but that didn’t work. I avoided the hospital and the clinic and everything because I was afraid. At 9 months I had a bad relapse, and later that day I actually went into labor. My daughter was born that day. A social worker called Child Services on me; I don’t blame her. I failed my daughter, and she was taken from me. She stays with my cousin, and I get to see her sometimes. She’s three now. I’m not sure if she knows who I am or not, and that really sucks.”

If asked how that experience impacts how you feel about this pregnancy, reply “After she was taken away, I quit cold turkey, for good this time. I haven’t touched anything since then. A few years went by. I met a new guy who is so good to me. We wanted to have a baby. But now that she, oh I hope it’s a she, is on the way, it’s overwhelming. I feel so nervous and guilty that the same thing is going to happen again. I’m staying clean for her, but I’m scared it’s not enough.”

If a plan is established to complete outdated prenatal care, reply “Okay, I can do that today.”

If the plan is well summarized prior to the end of the conversation, reply “Thank you all for listening and taking care of us.”

If the plan is not well summarized prior to the end of the conversation, reply “I’m overwhelmed by all of this information.”
