# Supplementary material for: A Multidisciplinary Standardized Patient Simulation for Using Trauma-Informed Care for Pregnant Patients
Source: MedEdPORTAL. 2024 Nov 26;20:11474. doi: 10.15766/mep_2374-8265.11474 (PMC11590754; doi:10.15766/mep_2374-8265.11474)
Supplement: Supplementary file 1 — Standardized Patient Case.docxStandardized Patient Guide.docxFacilitator Notes.docxFacilitator Education Guide.docxCase Flow.docxDebriefing Form.docxTrauma-Informed Care Presurvey.docxTrauma-Informed Care Postsurvey.docx [file mep_2374-8265.11474-s001.zip › A. Standardized Patient Case.docx]

Appendix A: Standardized Patient Case

*To be used during Standardized Patient training.*

Date: 10/11/2023

Primary Case Author: Kelly S Gibson MD

Secondary Case Author: Danielle Olson MD

Standardized Patient Educator: Kelly S Gibson MD

Name of Case: Prenatal Counseling After Prior Traumatic Experience

Name of Educational and/or Assessment Activity: Pre- and Post-Simulation Survey

Patient Name: Sadie Sadama

Chief Complaint: 25-year-old G2P1001 at 28w0d by last menstrual period presenting to establish prenatal care.

Most Likely Diagnosis and Differential With Rationale From History and/or Physical Exam:

- Patient with a prior traumatic experience impacting engagement with prenatal care in subsequent pregnancy
  - Supported by advanced gestational age noted on history and exam and lack of engagement with providers at the start of the conversation
- Patient with history of substance use in remission
  - Supported by lack of observed or reported withdrawal symptoms and lack of signs of current use
- Differential diagnosis
  - Ongoing substance use

Challenge Question: Can learners engage with a patient with a prior traumatic experience and build trust and rapport while ensuring a plan to provide the medical and obstetric standard of care?

Domains: Check all that apply

- Professionalism

X Communication and Interpersonal Skills

- Medical History
- Physical Exam
- Shared Decision-Making

X Patient Education

- Clinical Reasoning
- Documentation
- Handoff
- Presentation
- Other:

Type and Level of Learner:

- Anticipated learners include obstetric nurses, OB/Gyn residents, medical students, general OB/Gyn attendings, Maternal Fetal Medicine fellows and attendings, and Advanced Practice Providers.

Case Objectives: Please list specific objectives for each of the domains you have checked above:

1. Communication and Interpersonal Skills
   1. Collect and address the patient’s relevant history
   2. Empathize with patient’s past experiences as they relate to this pregnancy
   3. Praise patient’s efforts to maintain a healthy pregnancy
   4. Ask open-ended questions
   5. Demonstrate reflective listening
2. Patient Education
   1. Offer social work/other resources in a sensitive manner
   2. Ensure and explain a plan to get ultrasound and lab work completed
   3. Summarize the visit

| SETTING: outpatient, in patient, ED, home, nursing home, rehab, group, etc. | Outpatient exam room |
| --- | --- |
| PATIENT PROFILE: Information about the “patient” that helps select an SP and helps the learner get an understanding of them as a person. SP will know more information about the patient than learner will ever ask but allows SP to portray a fully developed patient personality. If none of the items below are particulars for the case, please write “all may be used.” | |
| Age range | 25-35 years old |
| Religious/spiritual background | All may be used. |
| Sex (e.g., male, female, intersex, transwoman, transman) | Female |
| Sexual orientation (e.g., heterosexual, lesbian, gay, bisexual, pansexual, queer, asexual) | Heterosexual |
| Gender expression (e.g., man, woman, genderqueer) | Woman |
| Race and ethnicity | All may be used. |
| Physical description (e.g., BMI, height range) | All may be used. |
| Physical limitations | None |
| Patient appearance (e.g., disheveled, hospital gown, business casual, casual) | Patient is well groomed, dressed in casual, clean clothing appropriate for the weather. |
| Moulage + location (e.g., none, bruises, scars, body piercing, tattoos) | No evidence of current or healed track marks. Tattoos may or may not be present. |
| Affect (e.g., pleasant, cooperative) | Initially withdrawn and disengaged, increasingly more engaged and pleasant/cooperative affect as counseling progresses. |
| Family group (e.g., who is family, who they live with) | Lives with long-term boyfriend, patient’s daughter lives with her aunt in the same city. |
| Education | Finished high school |
| Level of health literacy | Very limited |
| Employment, if any - present and past, noting any current stresses | She currently works as a cashier at a local gas station, used to work nights but was able to change to days after she reported safety concerns to her boss. She is happy with her job and enjoys her coworkers. |
| Home/homeless - type of dwelling, number of stories, owned or rented | Patient and her boyfriend live in a 2-bedroom apartment. The building is well maintained. Utilities are reliable. There are no pets in the home. |
| Financial situation - any current stresses | No current financial stressors. Patient and her boyfriend live together and share finances. Her job is secure, he has stable employment as well as a construction worker. |
| Insurance status (e.g., un/under/insured, public/private, HMO/PPO) | Enrolled in Medicaid prior to pregnancy. |
| Habits (i.e., diet, exercise, caffeine, smoking, alcohol, drugs) | Maintains balanced diet with rare fast food, walks daily, one cup of half-caffeine coffee in the mornings at work, no current alcohol or drug use, never smoker. |
| Activities (i.e., hobbies, sports, clubs, friends) | Enjoys watching movies and going for walks with her boyfriend. |
| Typical day - what is the usual daily routine | - Wakes up at 7 AM and eats breakfast while getting ready for work - Works first shift at the gas station, generally finishes work at 5 unless she needs to stay for inventory which occurs once per month - Gets home and takes a shower and begins preparing dinner - Dinner usually occurs between 7-7:30 PM, after which her boyfriend does the dishes while she sits and relaxes - Her and her boyfriend generally watch a few episodes of a TV show - Both go to bed around 11 PM |

| CASE INFORMATION | |
| --- | --- |
| Chief Concern: What the patient will say when greeted by the student. The patient’s primary reason for seeking medical care often stated in their own words. | “I’m about 6 or 7 months pregnant and I was reading online that I need bloodwork and an ultrasound.” |
| Additional Concerns: Other, if any, concerns the patient has today (i.e., symptoms, requests, expectations, etc.) that will become part of set agenda. | - Patient is approximately 15 minutes late for appointment - No complaints or symptoms today |
| THE PATIENT’S STORY: The SP will be asked to tell their symptom story and the personal and emotion impact for each of their concerns. You will want to write this in the patient’s voice. The symptom story should be able to answer this question: “Tell me more about [chief concern/additional concern], starting at the beginning and bringing me up to now.”  The personal context should be able to answer questions concerning the broader personal/psychosocial context of symptoms, especially the patient’s beliefs/attributions.  The emotional context should be able to ask how are you doing with this, how does this make you feel, how has this affected you emotionally? IMPACT: How has this affected your life? How has this been for your family? | “I haven’t come in yet because I have just been really busy and nervous about stuff.”  “I don’t want to go through all that again.”  The below is not volunteered, only given if providers ask and have established rapport.  “My last pregnancy, I was still using. I tried really hard to get on Subutex, but it didn’t work. I tried going cold turkey but that didn’t work. I avoided the hospital and the clinic and everything because I was afraid. At 9 months I had a bad relapse, and later that day I actually went into labor. My daughter was born that day. A social worker called Child Services on me; I don’t blame her. I failed my daughter, and she was taken from me. She stays with my cousin, and I get to see her sometimes. She’s three now. I’m not sure if she knows who I am or not, and that really sucks.”  “After she was taken away, I quit cold turkey, for good this time. I haven’t touched anything since then. A few years went by. I met a new guy who is so good to me. We wanted to have a baby. But now that she, oh I hope it’s a she, is on the way, it’s overwhelming. I feel so nervous and guilty that the same thing is going to happen again. I’m staying clean for her, but I’m scared it’s not enough.” |
| HISTORY OF PRESENT ILLNESS: Although some of the HPI will be given in the patient’s symptom story, the learners will expand the story during the direct question section. Below, describe the detailed history, usually about the chief concern, which the student must develop in order to make a useful assessment of the problem: | |
| Onset (when; gradual or sudden) | Her last menstrual period was approximately 28 weeks ago. |
| Setting (what was going on or where was patient when symptoms first noticed?) | Not applicable |
| Duration (how long) | Not applicable |
| Time relationships (frequency, constant or intermittent) | Not applicable |
| Location | Not applicable |
| Radiation | Not applicable |
| Quality | Not applicable |
| Amount | Not applicable |
| Aggravated by what | Not applicable |
| Relieved by what | Not applicable |
| Associated with what | Not applicable |
| Attitude (what does the patient think is the problem, and how do they feel about it) | Patient is cautiously excited about pregnancy, states it was planned and her partner is supportive. |
| Overall course | No complications to date. |
| REVIEW OF SYSTEMS: Significant positives and negatives | |
| - Negative: Vaginal bleeding - Negative: Contractions/cramping - Negative: Leaking of amniotic fluid/vaginal discharge - Negative: Decreased fetal movement - Negative: Withdrawal symptoms (yawning, irritability, diarrhea, vomiting, increased sweating, myalgias, etc.) | - No pertinent positive symptoms |
|  |  |
|  |  |
|  |  |
|  |  |
| Past medical history |  |
| Medication allergies (name and reaction) | No known medication allergies |
| Environmental allergies (name and reaction) | No known environmental allergies |
| Illnesses | No acute/chronic illnesses |
| Vaccinations | Up to date |
| Surgeries | No previous surgeries |
| Accidents/injuries/trauma | Broken arm as a teenager, set and healed without complication, no surgery required. Received oxycodone prescription for pain and developed subsequent addiction. |
| Hospitalization | One prior hospitalization for birth of first child, no records available to review from this hospitalization. |
|  | |
| Inclusive sexual and reproductive history | |
| Sexual practices  Sexual partners  Protection: Use of safer sex practices  Use of birth control if appropriate  Risk of intimate partner violence | - Heterosexual - One long-term male partner, boyfriend - Does not use protection - History of OCP use, no current/recent contraception - Negative screen for intimate partner violence, feels safe with her boyfriend |
| OB/GYN history | Age of onset of menses: 13 years old  Age of menopause: Not applicable  Number of pregnancies: 1 previous, currently pregnant  Number of live births: 1 prior full term vaginal delivery  Number of miscarriages: None  Number of abortions: None |
| Medications | Prescription/dose/reason   - No prescription medications   Over the counter/dose/reason   - Rare OTC acetaminophen (500mg) for headaches   Herbs/supplements/dose/reason   - Daily prenatal vitamin, OTC   Other:   - No additional medications or substances |
| Immunizations | X Tetanus – administered 3 years ago with pregnancy  X Flu – received annual dose through her job  X Hepatitis – received all childhood vaccines   - Pneumovax – not applicable   X HPV – received full course   - Other |
| Tobacco products:   - Cigarettes - Cigar - Pipe - Chew - E-cigarettes | X Never   - Past - year started/year quit - Current   - Quantity   - # of years |
| Alcohol   - Beer   X Wine   - Liquor - Other | - Never   X Past - year started/year quit   - - Rare glass of wine for special occasions   - Discontinued when attempting pregnancy   - Started at 22 years old - Current   - Quantity   - # of years |
| Drugs   - Weed - Cocaine   X Heroin   - Meth - IV - Inhalants   X Other   - - Oxycodone | - Never   X Past - year started/year quit   - Prior opioid use, oral pills and insufflation - Started with prescription for pain, developed opioid use disorder after. - Used pills for 3-4 years before switching primarily to insufflation, never used IV - Used for 7 years total, stopped 3 years ago - Current   - Quantity   - # of years |
| Diet (describe) | Strives to maintain balanced diet, has increased protein intake and decreased sugary foods since she found out about the pregnancy. Eats fast food approximately once per week. |
| Exercise (describe) | Spends the majority of her time standing and walking at work. Will most often go for short walks on her days off from work. |
| List any other important social history or information important to this case | Partner from her last pregnancy also used drugs, frequently supplied her with the drugs she used. He was never physically, sexually, or emotionally abusive, but he did not support her desire to become sober after their daughter was born. He is no longer involved in the patient’s or the daughter’s life and the patient has not been in contact with him in years. |
| Family history |  |
| Mother, father, siblings, grandparents, and other significant findings | Non-contributory |
| Physical Exam - List exam maneuvers expected for this case and any abnormal findings that SP will simulate. (tenderness, hyper-hypo reflex, rebound, weakness, etc.)  No abnormal findings elicited on physical exam maneuvers |  |
| PHYSICAL EXAM FINDINGS | |
| 1. Written in layperson’s terms | Pregnant female patient seated alone in an outpatient exam room. No acute distress, initially withdrawn and disengaged in conversation. Appears nervous, fidgeting. No abnormal physical exam findings aside from gravid uterus. |
| 1. General appearance - affect, appearance, position of patient at opening (i.e., sitting, lying down, holding abdomen, etc.) | Patient is initially sitting on chair in exam room, leaning back and looking down rather than at the provider. As the provider builds rapport, she will become more engaged and will exhibit good eye contact. Patient is well groomed, clothing is clean. |
| 1. Vital signs | BP 115/70, HR 90, RR 16, T 36.9°C, BMI 23 |
| 1. Specific findings and affect | - Affect will initially be flat, and patient will be disengaged, as the participants build rapport the affect will become more appropriate, and the patient will become more engaged - Gravid uterus with fundus above umbilicus, measuring approximately 28 weeks - Fetal heart tones 150 beats per minute |
| 1. Response to certain physical movements | Not applicable, no abnormal responses. |
|  |  |
| DIAGNOSIS AND DIFFERENTIAL |  |
| Diagnosis with support from positive and negative history and PE findings | Patient with prior traumatic experience impacting engagement with prenatal care in subsequent pregnancy   - Supported by advanced gestational noted on exam and lack of engagement with providers initially   Patient with history of substance use in remission   - Supported by lack of observed or reported withdrawal symptoms and lack of signs of current use |
| Differential with support from positive and negative history and PE findings | - History of substance use currently in remission - Ongoing substance use |
|  |  |
| MANAGEMENT OR DIAGNOSTIC PLAN | - Build rapport to ensure patient engagement in care - Assess patient’s understanding and agreement with the plan - Consultation with social work/similar entity - Schedule for obstetric ultrasound - Obtain baseline pregnancy blood/urine tests - Offer but do not demand toxicology screen - Schedule follow up visits for prenatal care |
|  |  |
| PROFESSIONALISM ISSUES OR CHALLENGES | Providers may profile patient and assume that this pregnancy was unplanned/undesired, that patient is still using substances, that there is no paternal involvement in this pregnancy, etc. If these things are assumed, the patient will be very offended, and the rapport will be significantly damaged. |
